# Supplementary material for: A comparation of dexmedetomidine and midazolam for sedation in patients with mechanical ventilation in ICU: A systematic review and meta-analysis
Source: PLoS One. 2023 Nov 14;18(11):e0294292. doi: 10.1371/journal.pone.0294292 (PMC10645332; doi:10.1371/journal.pone.0294292)

**A comparative evaluation of dexmedetomidine and midazolam in ICU mechanically ventilated patients: a systematic review and meta-analysis of randomized trials**

eTable S2: Evidence Profile Table

eTable S3: Sample Search Strategy

eFigure 1: Risk of bias graph

eFigure 2: Funnel Plot

eFigure 3. Meta-regression of the relationship between the mean age of participants and risk ratio for risk of delirium

eFigure 4. The Forest Plot of proportion of time at target sedation

eFigure 5. The Forest Plot of additional sedatives use

eFigure 6. The Forest Plot of Tachycardia

eFigure 7. The Forest Plot of Hypertension

eFigure 8. The Forest Plot of Hospital length of stay

eFigure 9. The Forest Plot of Hypotension Requiring Intervention

eFigure 10. The Forest Plot of Bradycardia Requiring Intervention

eFigure 11. The Forest Plot of ICU cost

eFigure 12. Intensive Care Unit Length of Stay Subgroup Analysis by Age of participants Forest Plot

eFigure 13. Intensive Care Unit Length of Stay Subgroup Analysis by Duration of sedation Forest Plot

eFigure 14. Intensive Care Unit Length of Stay Subgroup Analysis by APACHE II score Forest Plot

eFigure 15. Intensive Care Unit Length of Stay Subgroup Analysis by region Forest Plot

eFigure 16. Intensive Care Unit Length of Stay Sensitivity Analysis by risk of bias Forest Plot

eFigure 17. Intensive Care Unit Length of Stay Sensitivity Analysis by sample size Forest Plot

eFigure 18. Delirium Subgroup Analysis by Age of participants Forest Plot

eFigure 19. Delirium Subgroup Analysis by Duration of sedation Forest Plot

eFigure 20. Delirium Subgroup Analysis by APACHE II score Forest Plot

eFigure 21. Delirium Subgroup Analysis by region Forest Plot

eFigure 22. Delirium Sensitivity Analysis by risk of bias Forest Plot

eFigure 23. Delirium Sensitivity Analysis by sample size Forest Plot

eFigure 24. Duration of Mechanical Ventilation Subgroup Analysis by Age of participants Forest Plot

eFigure 25. Duration of Mechanical Ventilation Subgroup Analysis by Duration of sedation Forest Plot

eFigure 26. Duration of Mechanical Ventilation Subgroup Analysis by APACHE II score Forest Plot

eFigure 27. Duration of Mechanical Ventilation Subgroup Analysis by region Forest Plot

eFigure 28. Duration of Mechanical Ventilation Sensitivity Analysis by risk of bias Forest Plot

eFigure 29. Duration of Mechanical Ventilation Sensitivity Analysis by sample size Forest Plot

eFigure 30. Bradycardia Subgroup Analysis by Age of participants Forest Plot

eFigure 31. Bradycardia Subgroup Analysis by Duration of sedation Forest Plot

eFigure 32. Bradycardia Subgroup Analysis by APACHE II score Forest Plot

eFigure 33. Bradycardia Subgroup Analysis by region Forest Plot

eFigure 34. Bradycardia Sensitivity Analysis by risk of bias Forest Plot

eFigure 35. Bradycardia Sensitivity Analysis by sample size Forest Plot

eFigure 36. Hypotension Subgroup Analysis by Age of participants Forest Plot

eFigure 37. Hypotension Subgroup Analysis by Duration of sedation Forest Plot

eFigure 38. Hypotension a Subgroup Analysis by APACHE II score Forest Plot

eFigure 39. Hypotension Subgroup Analysis by region Forest Plot

eFigure 40. Hypotension Sensitivity Analysis by risk of bias Forest Plot

eFigure 41. Hypotension Sensitivity Analysis by sample size Forest Plot

eFigure 42. Mortality Subgroup Analysis by Age of participants Forest Plot

eFigure 43. Mortality Subgroup Analysis by Duration of sedation Forest Plot

eFigure 44. Mortality Subgroup Analysis by APACHE II score Forest Plot

eFigure 45. Mortality Subgroup Analysis by region Forest Plot

eFigure 46. Mortality Sensitivity Analysis by risk of bias Forest Plot

eFigure 47. Mortality Sensitivity Analysis by sample size Forest Plot

**eTable S2:Evidence Profile Table**

| **DEX compared to MDZ for ICU mechanically ventilated patients** | | | | | | | | | | | |
| --- | --- | --- | --- | --- | --- | --- | --- | --- | --- | --- | --- |
| **Certainty assessment** | | | | | | | **Summary of findings** | | | | |
| **Participants**  **(studies)**  **Follow-up** | **Risk of bias** | **Inconsistency** | **Indirectness** | **Imprecision** | **Publication bias** | **Overall certainty of evidence** | **Study event rates (%)** | | **Relative effect**  **(95% CI)** | **Anticipated absolute effects** | |
|  |  |  |  |  |  |  | **With MDZ** | **With DEX** |  | **Risk with MDZ** | **Risk difference with DEX** |
| **ICU length of stay** | | | | | | | | | | | |
| 1779  (13 RCTs) | not serious | serious^a^ | not serious | not serious | none | ⨁⨁⨁◯  Moderate | 799 | 980 | - |  | MD **2.25 SD lower**  (2.94 lower to 1.57 lower) |
| **Delirium** | | | | | | | | | | | |
| 1758  (12 RCTs) | not serious | serious^b^ | not serious | not serious | none | ⨁⨁⨁◯  Moderate | 283/799 (35.4%) | 270/959 (28.2%) | **RR 0.63**  (0.50 to 0.81) | 354 per 1, 000 | **131 fewer per 1, 000**  (from 177 fewer to 67 fewer) |
| **Duration of mechanical ventilation** | | | | | | | | | | | |
| 1433  (10 RCTs) | serious^c^ | very serious^d^ | not serious | not serious | none | ⨁◯◯◯  Very low | 655 | 778 | - |  | MD **0.83 SD lower**  (1.24 lower to 0.43 lower) |
| **Bradycardia** | | | | | | | | | | | |
| 1578  (10 RCTs) | not serious | not serious | not serious | not serious | none | ⨁⨁⨁⨁  High | 48/702 (6.8%) | 181/876 (20.7%) | **RR 2.21**  (1.31 to 3.73) | 68 per 1, 000 | **83 more per 1, 000**  (from 21 more to 187 more) |
| **Hypotension** | | | | | | | | | | | |
| 1427  (9 RCTs) | not serious | serious^e^ | not serious | serious^f^ | none | ⨁⨁◯◯  Low | 115/651 (17.7%) | 225/776 (29.0%) | **RR 1.44**  (0.87 to 2.38) | 177 per 1, 000 | **78 more per 1, 000**  (from 23 fewer to 244 more) |
| **Mortality** | | | | | | | | | | | |
| 1662  (11 RCTs) | not serious | not serious | not serious | serious^g^ | none | ⨁⨁⨁◯  Moderate | 131/741 (17.7%) | 172/921 (18.7%) | **RR 1.02**  (0.83 to 1.25) | 177 per 1, 000 | **4 more per 1, 000**  (from 30 fewer to 44 more) |
| **ICU cost** | | | | | | | | | | | |
| 1013  (3 RCTs) | not serious | not serious | not serious | very serious^n^ | none | ⨁⨁◯◯  Low | 568 | 445 | - |  | MD **4.29 lower**  (7.16 lower to 1.42 lower) |
| **Hypotension Requiring Intervention** | | | | | | | | | | | |
| 609  (5 RCTs) | not serious | not serious | not serious | serious^n^ | none | ⨁⨁⨁◯  Moderate | 78/363 (21.5%) | 34/246 (13.8%) | **RR 1.45**  (0.70 to 3.00) | 215 per 1, 000 | **97 more per 1, 000**  (from 64 fewer to 430 more) |
| **Bradycardia Requiring Intervention** | | | | | | | | | | | |
| 428  (2 RCTs) | not serious | not serious | not serious | very serious^n^ | none | ⨁⨁◯◯  Low | 12/273 (4.4%) | 2/155 (1.3%) | **RR 1.98**  (0.14 to 28.72) | 44 per 1, 000 | **43 more per 1, 000**  (from 38 fewer to 1, 000 more) |
| **Proportion of time at target sedation** | | | | | | | | | | | |
| 972  (4 RCTs) | not serious | not serious | not serious | very serious^n^ | none | ⨁⨁◯◯  Low | 428 | 544 | - |  | MD **0.91 higher**  (3.9 lower to 5.72 higher) |
| **Use of additional sedatives** | | | | | | | | | | | |
| 1149  (6 RCTs) | serious^i^ | not serious | not serious | serious^n^ | none | ⨁⨁◯◯  Low | 307/633 (48.5%) | 227/516 (44.0%) | **RR 1.04**  (0.86 to 1.26) | 485 per 1, 000 | **19 more per 1, 000**  (from 68 fewer to 126 more) |
| **Hospital length of stay** | | | | | | | | | | | |
| 747  (4 RCTs) | not serious | very serious^j^ | not serious | very serious^n^ | none | ⨁◯◯◯  Very low | 370 | 377 | - |  | MD **0.11 higher**  (5.9 lower to 6.11 higher) |
| **Tachycardia** | | | | | | | | | | | |
| 1039  (4 RCTs) | not serious | not serious | not serious | very serious^n^ | none | ⨁⨁◯◯  Low | 117/458 (25.5%) | 125/581 (21.5%) | **RR 0.73**  (0.51 to 1.04) | 255 per 1, 000 | **69 fewer per 1, 000**  (from 125 fewer to 10 more) |
| **Hypertension** | | | | | | | | | | | |
| 1016  (3 RCTs) | not serious | not serious | not serious | very serious^n^ | none | ⨁⨁◯◯  Low | 159/446 (35.7%) | 111/570 (19.5%) | **RR 0.98**  (0.74 to 1.29) | 357 per 1, 000 | **7 fewer per 1, 000**  (from 93 fewer to 103 more) |

**CI:** confidence interval; **MD:** mean difference; **RR:** risk ratio

Explanations

a. Heterogeneity: Tau² = 0.68; Chi² = 25.61, df = 12 (P = 0.01); I² = 53%

b. Heterogeneity: Tau² = 0.07; Chi² = 23.66, df = 11 (P = 0.01); I² = 53%

c. 5/10 trials at low ROB. Low ROB MD -0.15 (95% CI -0.36 to 0.05), high ROB MD -1.57 (95% CI -2.17 to -0.97); I2 = 94.8% (P <0.01)

d. Heterogeneity: Tau² = 0.25; Chi² = 324.70, df = 9 (P < 0.00001); I² = 97%

e. Heterogeneity: Chi² = 22.05, df = 8 (P = 0.005); I² = 64%

f. TSA with AIS smaller than RIS (1427/2358), no boundaries crossed.

g. TSA with AIS smaller than RIS (914/138437), no boundaries crossed.

h. Relatively small overall sample size with few events

i. 3/6 trials at low ROB.

j. Heterogeneity: Tau² = 29.59; Chi² = 25.43, df = 3 (P < 0.0001); I² = 88%

**eTable S3:Sample Search Strategy.**

**Pubmed**

| **Search** | **Query** | **Items found** |
| --- | --- | --- |
| #1 | (((((((((Midazolam) OR (Midazolam Maleate)) OR (Maleate, Midazolam)) OR (Dormicum)) OR (Versed)) OR (Midazolam Hydrochloride)) OR (Hydrochloride, Midazolam)) OR (Ro 21-3981)) OR (Ro 21 3981)) OR (Ro 213981) | 17002 |
| #2 | ((((((Dexmedetomidine) OR (MPV-1440)) OR (MPV 1440)) OR (MPV1440)) OR (Precedex)) OR (Dexmedetomidine Hydrochloride)) OR (Hydrochloride, Dexmedetomidine) | 8233 |
| #3 | (((Intensive Care Units) OR (Intensive Care Unit)) OR (Unit, Intensive Care)) OR (ICU Intensive Care Units) | 239338 |
| #4 | (((((((Respiration, Artificial) OR (Artificial Respiration)) OR (Artificial Respirations)) OR (Respirations, Artificial)) OR (Ventilation, Mechanical)) OR (Mechanical Ventilations)) OR (Ventilations, Mechanical)) OR (Mechanical Ventilation) | 123684 |
| #5 | ((randomized controlled trial[Publication Type]) OR (randomized[Title/Abstract])) OR (placebo[Title/Abstract]) | 977624 |
| #6 | ((((#1) AND (#2)) AND (#3)) AND (#4)) AND (#5) | 40 |

**Embase**

| **Search** | **Query** | **Items found** |
| --- | --- | --- |
| #1 | 'midazolam'/exp | 56,432 |
| #2 | midazolam:ti,ab,kw OR 'midazolam maleate':ti,ab,kw OR 'maleate,midazolam':ti,ab,kw OR dormicum:ti,ab,kw OR versed:ti,ab,kw OR 'midazolam hydrochloride':ti,ab,kw OR 'hydrochloride,midazolam':ti,ab,kw OR 'ro 21-3981':ti,ab,kw OR 'ro 21 3981':ti,ab,kw OR 'ro 213981':ti,ab,kw | 24,132 |
| #3 | #1 OR #2 | 59,867 |
| #4 | 'dexmedetomidine'/exp | 16,519 |
| #5 | dexmedetomidine:ti,ab,kw OR 'mpv 1440':ti,ab,kw OR mpv1440:ti,ab,kw OR 'dexmedetomidine hydrochloride':ti,ab,kw OR precedex:ti,ab,kw OR 'hydrochloride, dexmedetomidine':ti,ab,kw | 11,299 |
| #6 | #4 OR #5 | 16,960 |
| #7 | 'intensive care unit'/exp | 265,304 |
| #8 | 'intensive care units':ti,ab,kw OR 'intensive care unit':ti,ab,kw OR 'unit, intensive care':ti,ab,kw OR 'icu intensive care units':ti,ab,kw | 212,339 |
| #9 | #7 or #8 | 328,073 |
| #10 | 'artificial ventilation'/exp | 268,915 |
| #11 | 'respiration,artificial':ti,ab,kw OR 'artificial respiration':ti,ab,kw OR 'artificial respirations':ti,ab,kw OR 'respirations, artificial':ti,ab,kw OR 'ventilation, mechanical':ti,ab,kw OR 'mechanical ventilations':ti,ab,kw OR 'ventilations, mechanical':ti,ab,kw OR 'mechanical ventilation':ti,ab,kw OR 'artificial ventilation':ti,ab,kw | 96,617 |
| #12 | #10 OR #11 | 282,594 |
| #13 | 'randomized controlled trial':ab,ti OR 'randomized':ab,ti OR 'placebo':ab,ti | 1078839 |
| #14 | #3AND #6 AND #9 AND #12AND#13 | 122 |

**Cochrane Library**

| **Search** | **Query** | **Items found** |
| --- | --- | --- |
| #1 | MeSH descriptor: [Midazolam] explode all trees | 3250 |
| #2 | (versed):ti,ab,kw OR (midazolam maleate):ti,ab,kw OR (dormicum):ti,ab,kw OR (midazolam):ti,ab,kw OR (ro 213981):ti,ab,kw (Word variations have been searched) | 10299 |
| #3 | #1 OR #2 | 10299 |
| #4 | MeSH descriptor: [Dexmedetomidine] explode all trees | 2227 |
| #5 | (mpv 1440):ti,ab,kw OR (precedex):ti,ab,kw OR (hydrochloride, dexmedetomidine):ti,ab,kw OR (dexmedetomidine):ti,ab,kw OR (dexmedetomidine hydrochloride):ti,ab,kw (Word variations have been searched) | 7201 |
| #6 | #4 or #5 | 7201 |
| #7 | MeSH descriptor: [Intensive Care Units] explode all trees | 4145 |
| #8 | (intensive care units):ti,ab,kw OR (intensive care unit):ti,ab,kw OR (unit, intensive care):ti,ab,kw OR (icu intensive care units):ti,ab,kw (Word variations have been searched) | 25265 |
| #9 | #7 or #8 | 25507 |
| #10 | MeSH descriptor: [Respiration, Artificial] explode all trees | 10301 |
| #11 | MeSH descriptor: [Ventilators, Mechanical] explode all trees | 301 |
| #12 | (mechanical ventilations):ti,ab,kw OR (ventilations, mechanical):ti,ab,kw OR (artificial ventilation):ti,ab,kw OR (artificial respiration):ti,ab,kw OR (respirations, artificial):ti,ab,kw (Word variations have been searched) | 20447 |
| #13 | #10OR#11OR#12 | 22738 |
| #14 | ("randomized controlled trial"):pt OR (randomized):ti,ab,kw OR (placebo):ti,ab,kw (Word variations have been searched) | 1283625 |
| #15 | #3AND#6AND#9AND#13AND#14 | 84 |

**Web of Science**

| **Search** | **Query** | **Items found** |
| --- | --- | --- |
| #1 | (((((((((TS=(Midazolam)) OR TS=(Midazolam Maleate)) OR TS=(Maleate, Midazolam)) OR TS=(Dormicum)) OR TS=(Versed)) OR TS=(Midazolam Hydrochloride)) OR TS=(Hydrochloride, Midazolam)) OR TS=(Ro 21-3981)) OR TS=(Ro 21 3981)) OR TS=(Ro 213981) | 32555 |
| #2 | ((((((TS=(Dexmedetomidine )) OR TS=(MPV-1440)) OR TS=(MPV 1440)) OR TS=(MPV1440)) OR TS=(Precedex)) OR TS=(Dexmedetomidine Hydrochloride)) OR TS=(Hydrochloride, Dexmedetomidine) | 11488 |
| #3 | (((TS=(Intensive Care Units)) OR TS=(Intensive Care Unit)) OR TS=(Unit, Intensive Care)) OR TS=(ICU Intensive Care Units) | 228616 |
| #4 | (((((((TS=(Mechanical Ventilation)) OR TS=(Respiration, Artificial)) OR TS=( Ventilations, Mechanical)) OR TS=(Artificial Respiration)) OR TS=(Artificial Respirations)) OR TS=(Respirations, Artificial)) OR TS=(Ventilation, Mechanical)) OR TS=(Mechanical Ventilations) | 132441 |
| #5 | (((TS=(randomized controlled trial)) OR TS=(randomized)) OR TS=(placebo)) OR TS=(RCT) | 1297075 |
| #6 | #1 AND #2 AND #3 AND #4 AND #5 | 106 |

**EBSCO**

| **Search** | **Query** | **Items found** |
| --- | --- | --- |
| #1 | TX Ro 21 3981 OR TX Versed OR TX Dormicum OR TX Midazolam Maleate OR TX Midazolam OR TX Maleate, Midazolam OR TX Ro 213981 OR TX Midazolam Hydrochloride OR TX Hydrochloride, Midazolam OR TX Ro 21-398 | 101,199 |
| #2 | TX Hydrochloride, Dexmedetomidine OR TX Dexmedetomidine OR TX MPV-1440 OR TX MPV 1440 OR TX MPV1440 OR TX Precedex OR TX Dexmedetomidine Hydrochloride | 5,145 |
| #3 | TX Unit, Intensive Care OR TX ICU Intensive Care Units OR TX Intensive Care Units OR TX Intensive Care Unit | 169,967 |
| #4 | TX Mechanical Ventilation OR TX Respiration, Artificial OR TX Artificial Respiration OR TX Artificial Respirations OR TX Respirations, Artificial OR TX Ventilation, Mechanical OR TX Mechanical Ventilations OR TX Ventilations, Mechanical | 39,923 |
| #5 | TX RCT OR TX randomized controlled trial OR TX placebo OR TX randomized | 778,797 |
| #6 | #1 AND #2 AND #3 AND #4AND#5 | 307 |

**Scopus**

| **Search** | **Query** | **Items found** |
| --- | --- | --- |
| #1 | ( TITLE-ABS-KEY ( ro 21-398 ) OR TITLE-ABS-KEY ( versed ) OR TITLE-ABS-KEY ( ro 21 3981 ) OR TITLE-ABS-KEY ( dormicum ) OR TITLE-ABS-KEY ( midazolam AND maleate ) OR TITLE-ABS-KEY ( midazolam ) OR TITLE-ABS-KEY ( maleate, AND midazolam ) OR TITLE-ABS-KEY ( ro 213981 ) OR TITLE-ABS-KEY ( midazolam AND hydrochloride ) OR TITLE-ABS-KEY ( hydrochloride, AND midazolam ) ) | 55415 |
| #2 | ( TITLE-ABS-KEY ( mpv1440 ) OR TITLE-ABS-KEY ( dexmedetomidine AND hydrochloride ) OR TITLE-ABS-KEY ( precedex ) OR TITLE-ABS-KEY ( hydrochloride, AND dexmedetomidine ) OR TITLE-ABS-KEY ( dexmedetomidine ) OR TITLE-ABS-KEY ( mpv-1440 ) OR TITLE-ABS-KEY ( mpv 1440 ) ) | 14660 |
| #3 | ( TITLE-ABS-KEY ( intensive AND care AND unit ) OR TITLE-ABS-KEY ( intensive AND care AND units ) OR TITLE-ABS-KEY ( icu AND intensive AND care AND units ) OR TITLE-ABS-KEY ( unit, AND intensive AND care ) ) | 283322 |
| #4 | ( TITLE-ABS-KEY ( ventilations, AND mechanical ) OR TITLE-ABS-KEY ( mechanical AND ventilation ) OR TITLE-ABS-KEY ( respiration, AND artificial ) OR TITLE-ABS-KEY ( artificial AND respiration ) OR TITLE-ABS-KEY ( artificial AND respirations ) OR TITLE-ABS-KEY ( respirations, AND artificial ) OR TITLE-ABS-KEY ( ventilation, AND mechanical ) OR TITLE-ABS-KEY ( mechanical AND ventilations ) ) | 116919 |
| #5 | ( TITLE-ABS-KEY ( randomized AND controlled AND trial ) OR TITLE-ABS-KEY ( randomized ) OR TITLE-ABS-KEY ( placebo ) OR TITLE-ABS-KEY ( rct ) ) | 1484593 |
| #6 | #1 AND #2 AND #3 AND #4AND #5 | 120 |

**eFigure 1: Risk of bias**


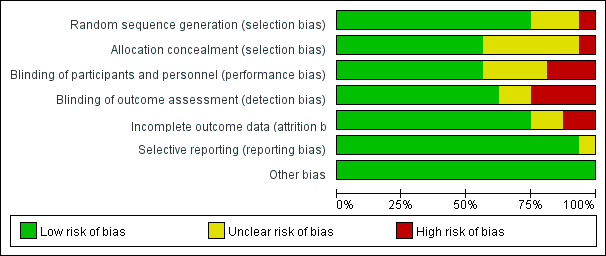

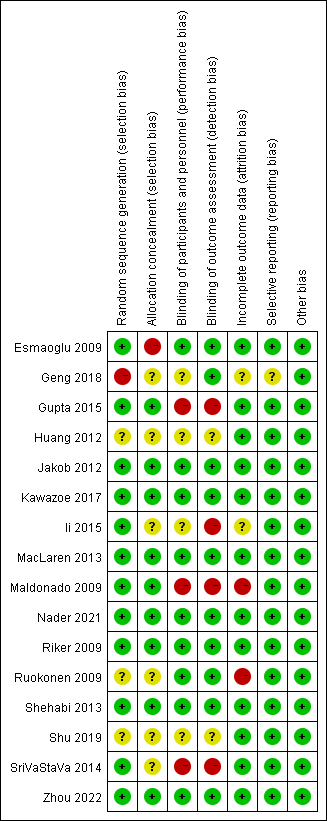


**eFigure 2: Funnel Plot**


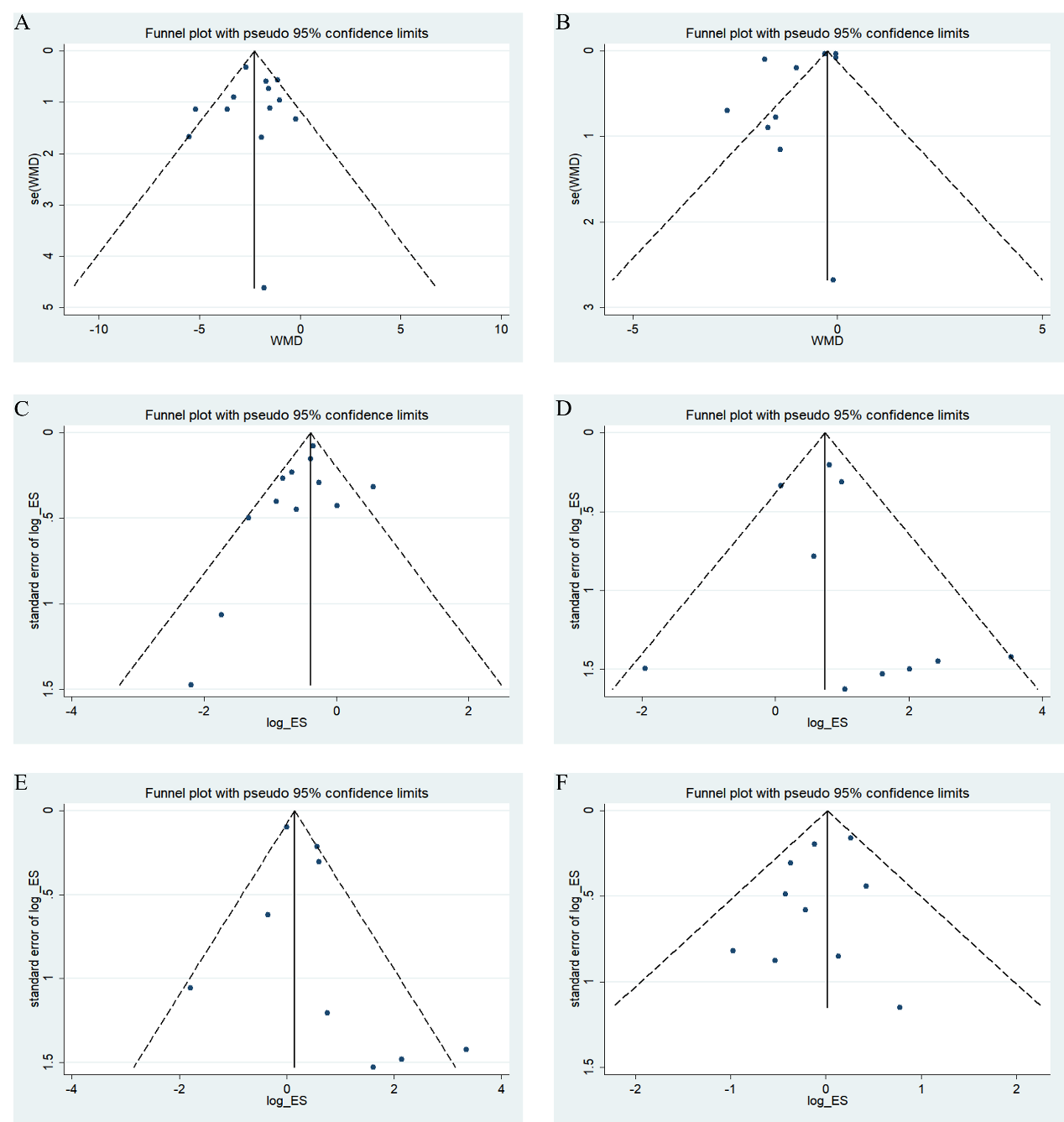


Explanations: A-Intensive Care Unit Length of Stay; B-Duration of Mechanical Ventilation; C‑Delirium. D-Bradycardia. E-Hypotension. F-Mortality.

**eFigure 3. Meta-regression of the relationship between the mean age of participants and risk ratio for risk of delirium**


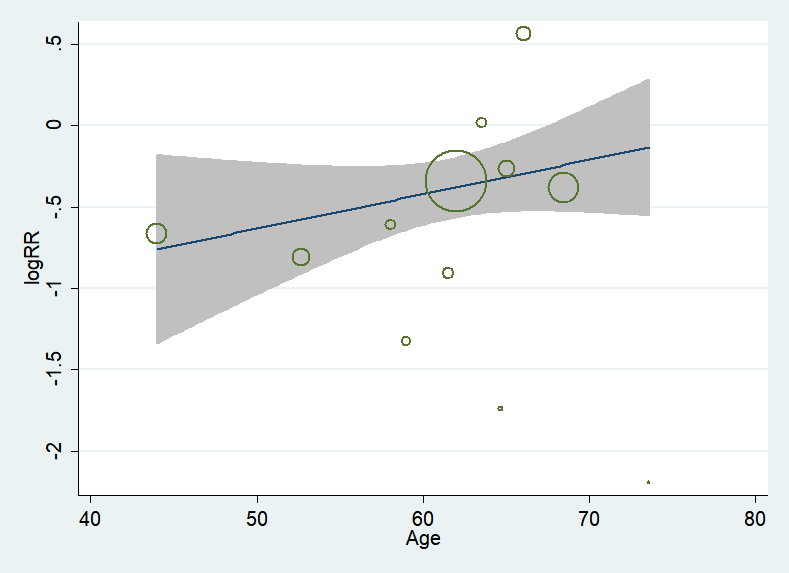


**eFigure 4. The Forest Plot of proportion of time at target sedation**

**eFigure 5. The Forest Plot of additional sedatives use**


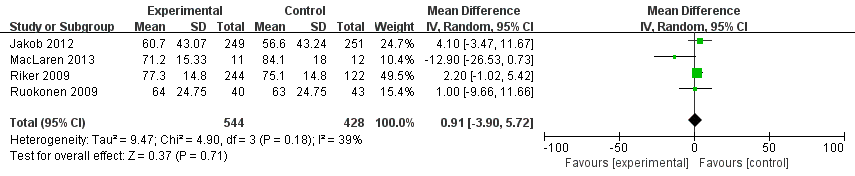

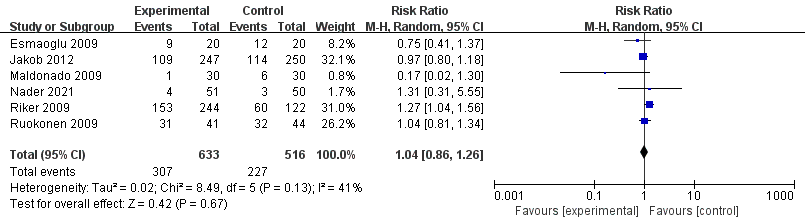


**eFigure 6. The Forest Plot of Tachycardia**


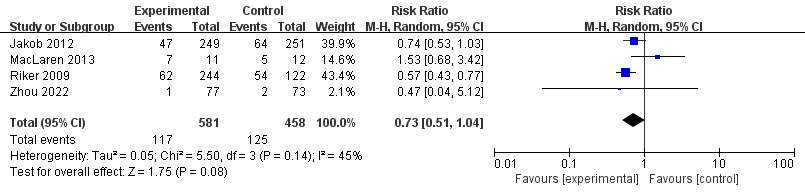


**eFigure 7. The Forest Plot of Hypertension**


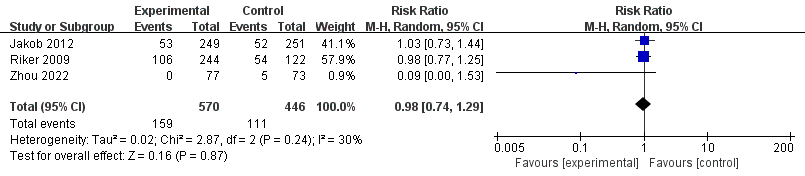


**eFigure 8. The Forest Plot of Hospital length of stay**


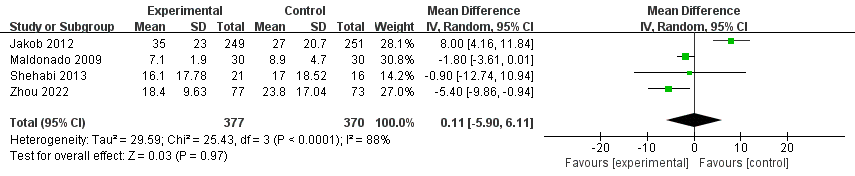


**eFigure 9. The Forest Plot of Hypotension Requiring Intervention**


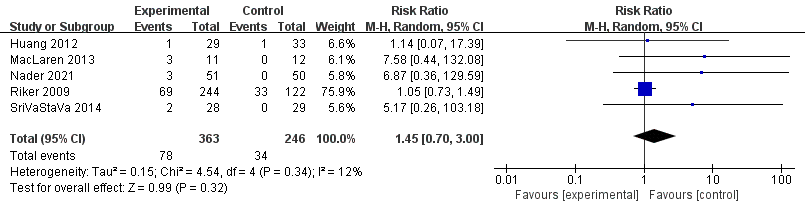


**eFigure 10. The Forest Plot of Bradycardia Requiring Intervention**


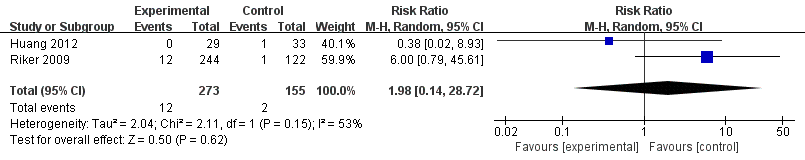


**eFigure 11. The Forest Plot of ICU cost**


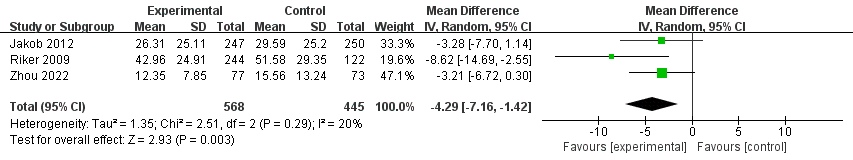


**eFigure 12. Intensive Care Unit Length of Stay Subgroup Analysis by Age of participants Forest Plot**


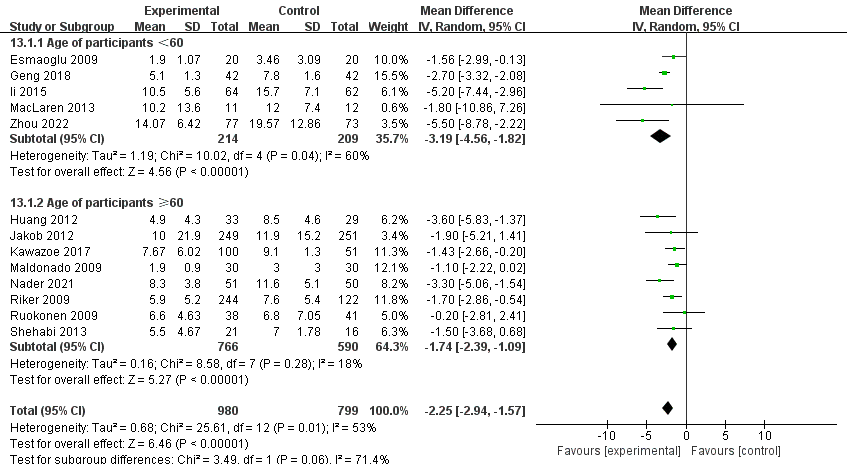


**eFigure 13. Intensive Care Unit Length of Stay Subgroup Analysis by Duration of sedation Forest Plot**


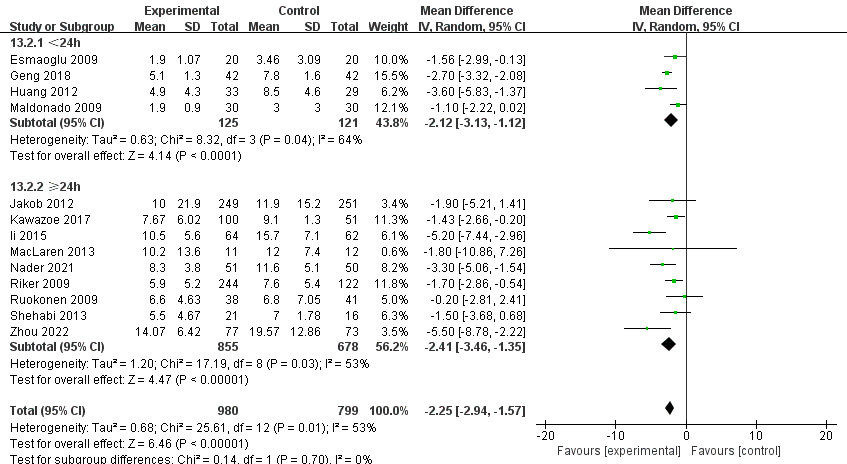


**eFigure 14. Intensive Care Unit Length of Stay Subgroup Analysis by APACHE II score Forest Plot**


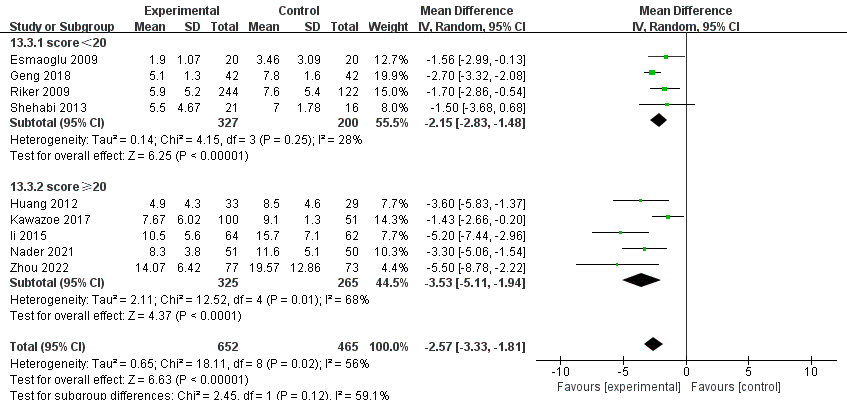


**eFigure 15. Intensive Care Unit Length of Stay Subgroup Analysis by region Forest Plot**


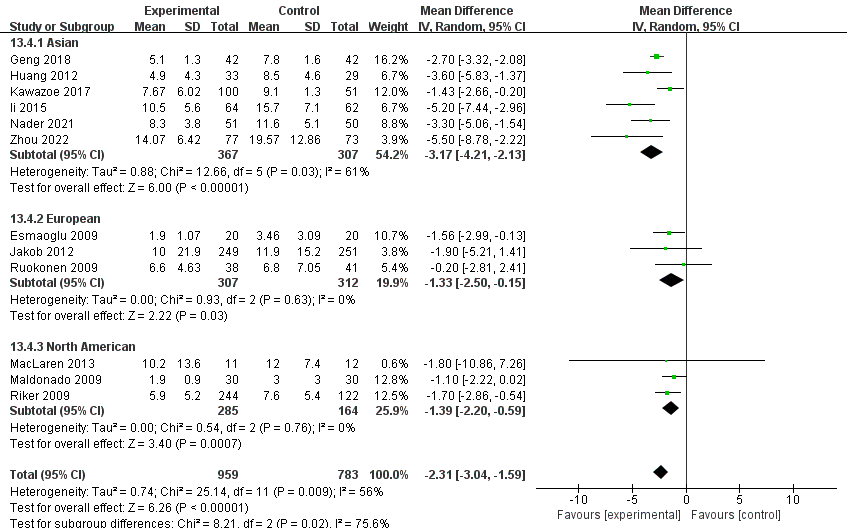


**eFigure 16. Intensive Care Unit Length of Stay Sensitivity Analysis by risk of bias Forest Plot**


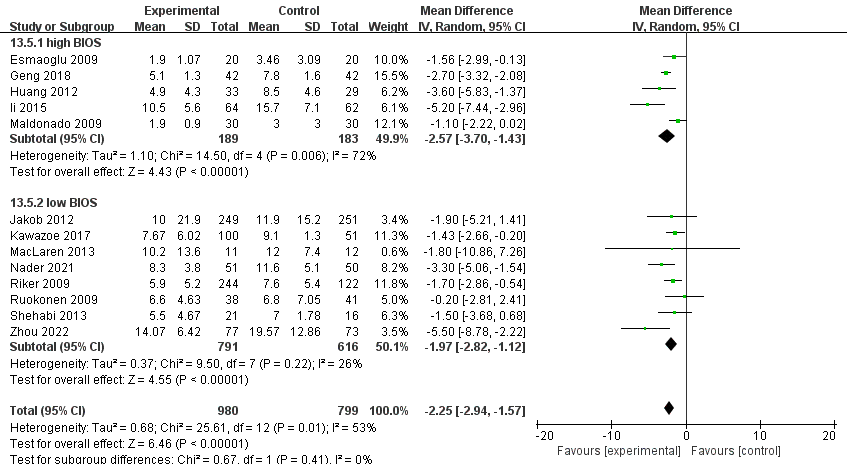


**eFigure 17. Intensive Care Unit Length of Stay Sensitivity Analysis by sample size Forest Plot**


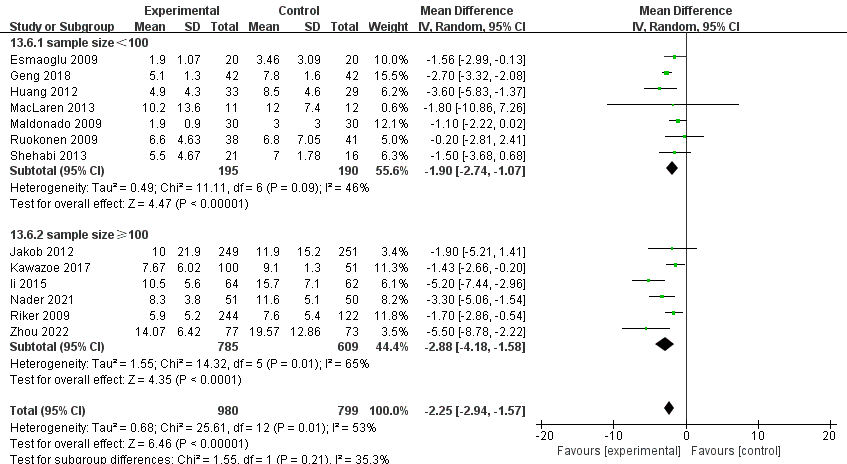


**eFigure 18. Delirium Subgroup Analysis by Age of participants Forest Plot**


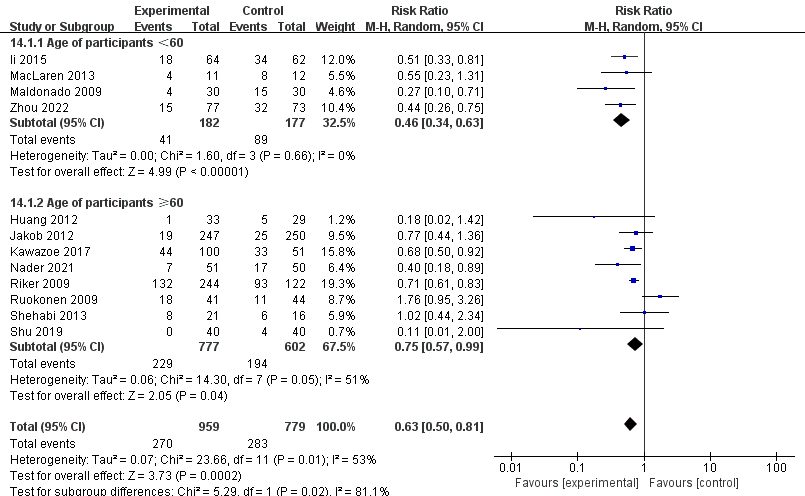


**eFigure 19. Delirium Subgroup Analysis by Duration of sedation Forest Plot**


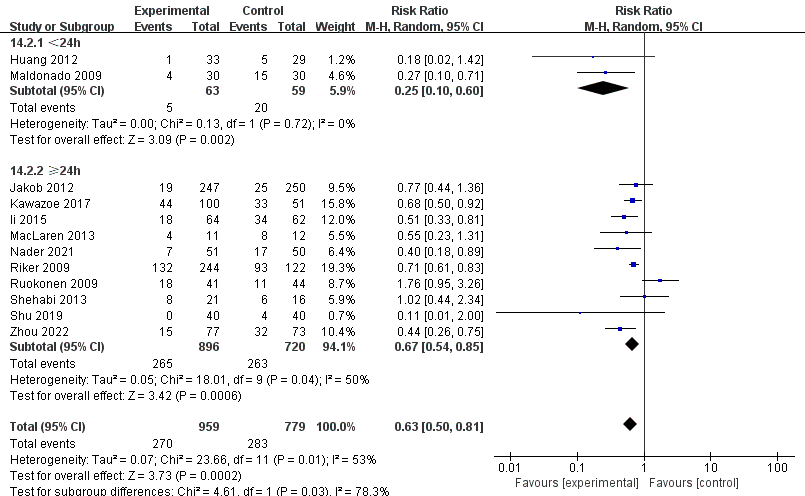


**eFigure 20. Delirium Subgroup Analysis by APACHE II score Forest Plot**


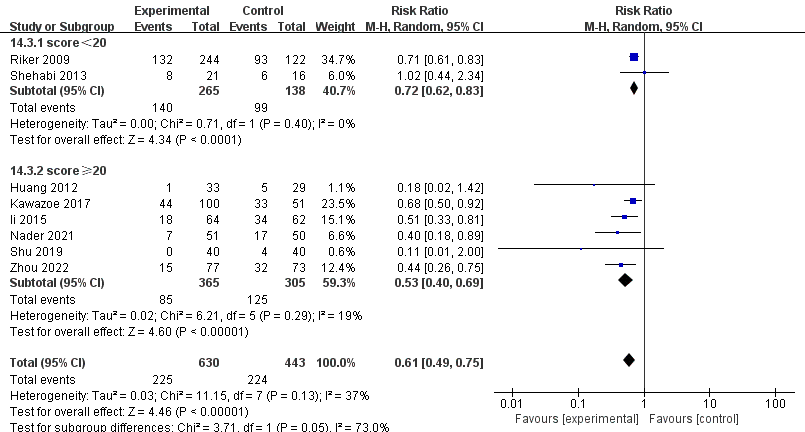


**eFigure 21. Delirium Subgroup Analysis by region Forest Plot**


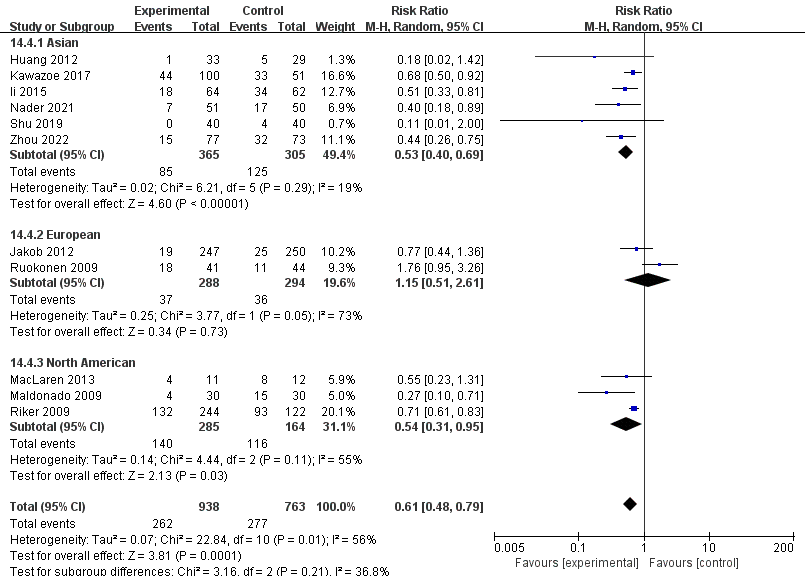


**eFigure 22. Delirium Sensitivity Analysis by risk of bias Forest Plot**


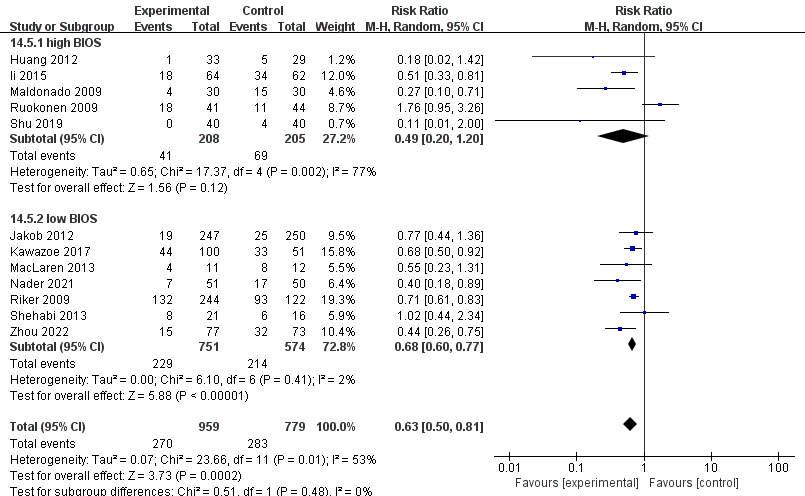


**eFigure 23. Delirium Sensitivity Analysis by sample size Forest Plot**


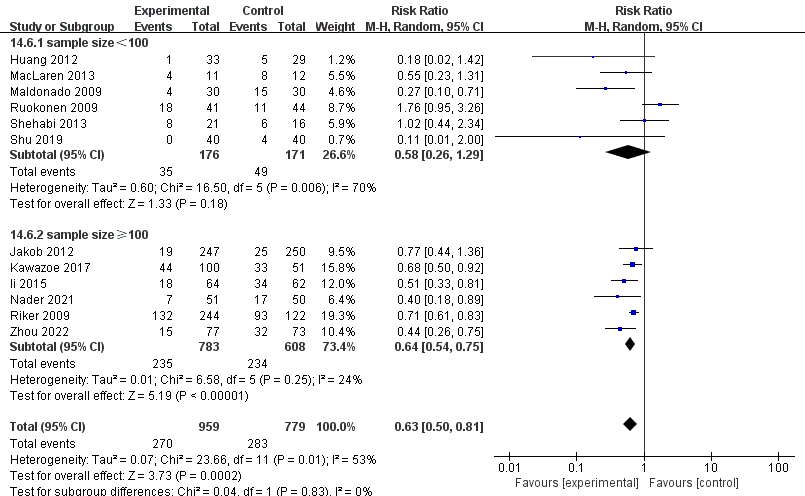


**eFigure 24. Duration of Mechanical Ventilation Subgroup Analysis by Age of participants Forest Plot**


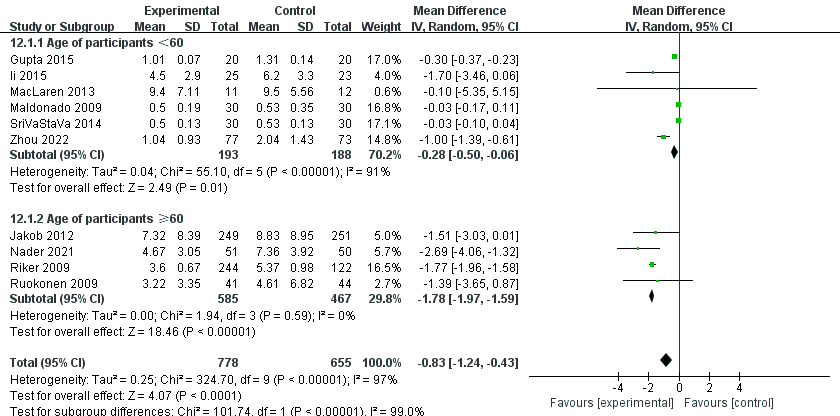


**eFigure 25. Duration of Mechanical Ventilation Subgroup Analysis by Duration of sedation Forest Plot**


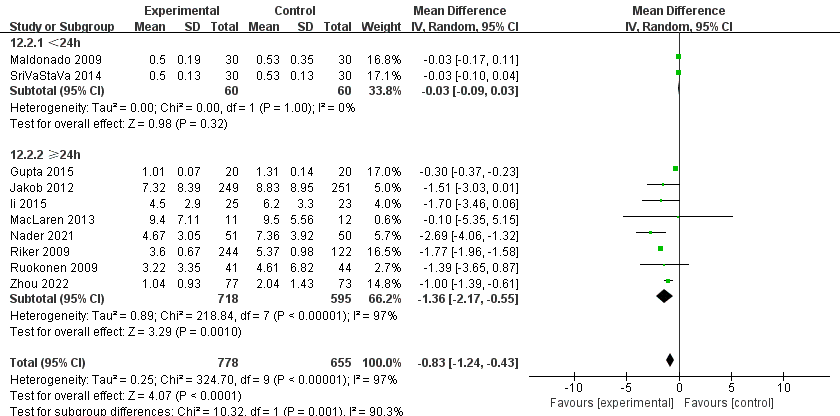


**eFigure 26. Duration of Mechanical Ventilation Subgroup Analysis by APACHE II score Forest Plot**


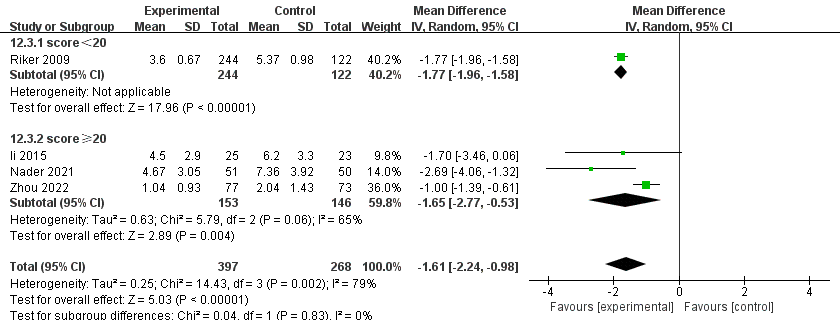


**eFigure 27. Duration of Mechanical Ventilation Subgroup Analysis by region Forest Plot**


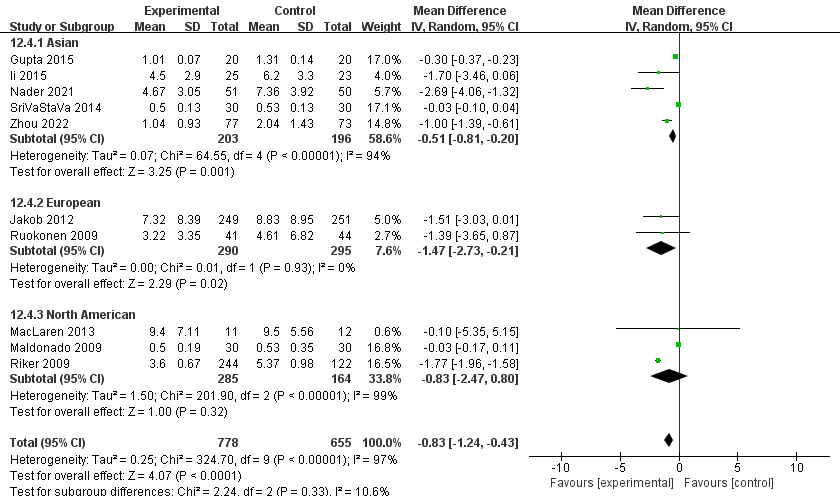


**eFigure 28. Duration of Mechanical Ventilation Sensitivity Analysis by risk of bias Forest Plot**


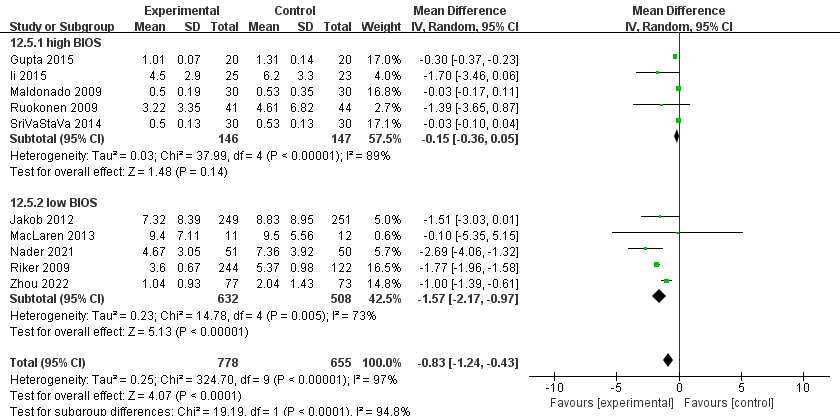


**eFigure 29. Duration of Mechanical Ventilation Sensitivity Analysis by sample size Forest Plot**


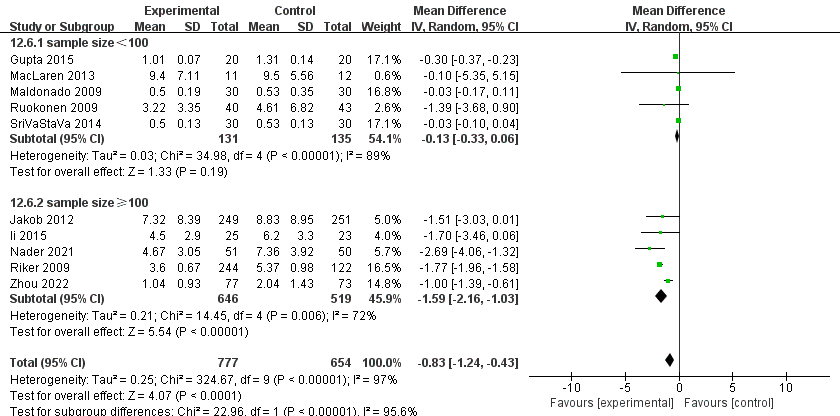


**eFigure 30.** **Bradycardia Subgroup Analysis by Age of participants Forest Plot**


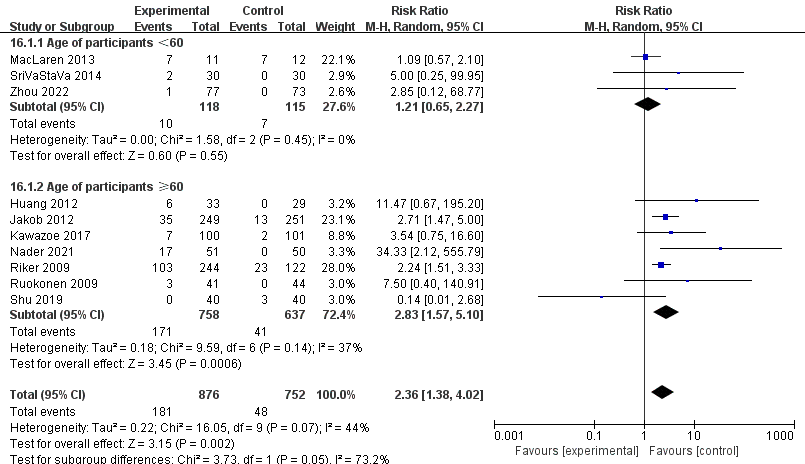


**eFigure 31. Bradycardia Subgroup Analysis by Duration of sedation Forest Plot**


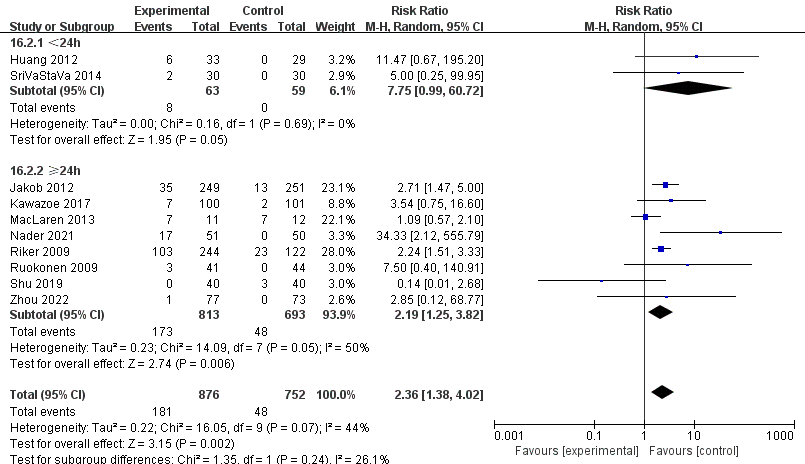


**eFigure 32.** **Bradycardia Subgroup Analysis by APACHE II score Forest Plot**


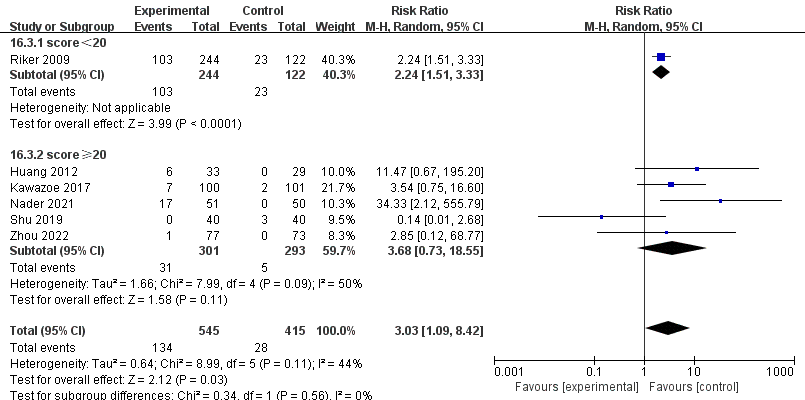


**eFigure 33. Bradycardia Subgroup Analysis by region Forest Plot**


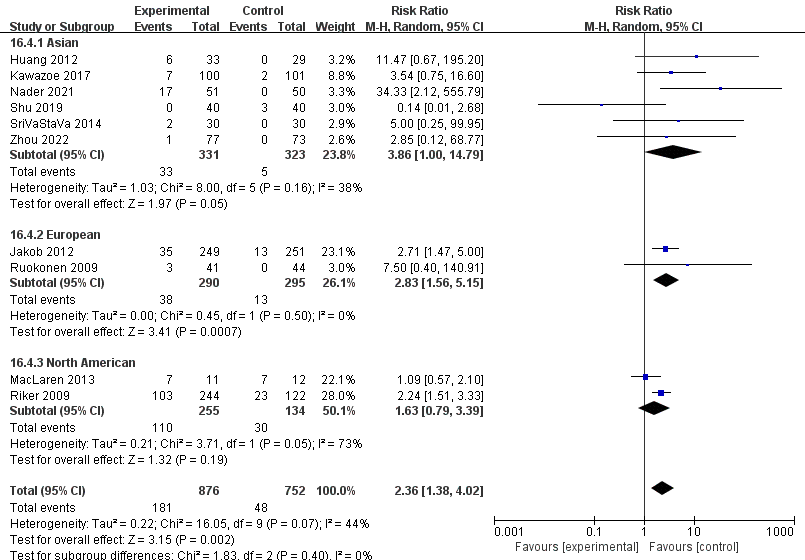


**eFigure 34. Bradycardia Sensitivity Analysis by risk of bias Forest Plot**


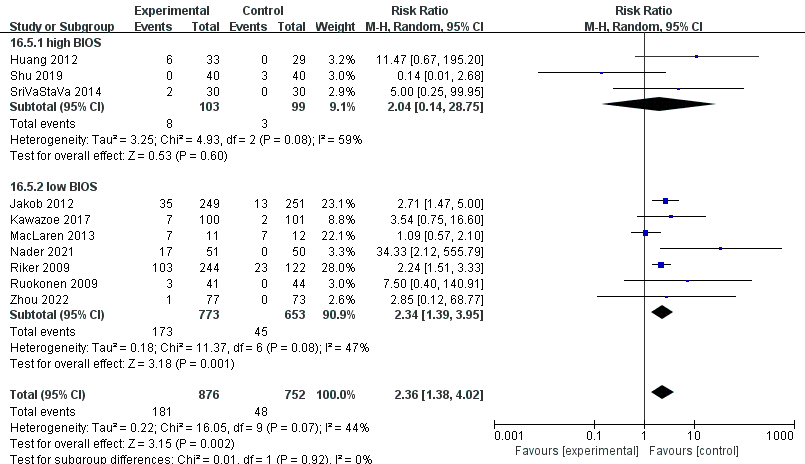


**eFigure 35. Bradycardia Sensitivity Analysis by sample size Forest Plot**


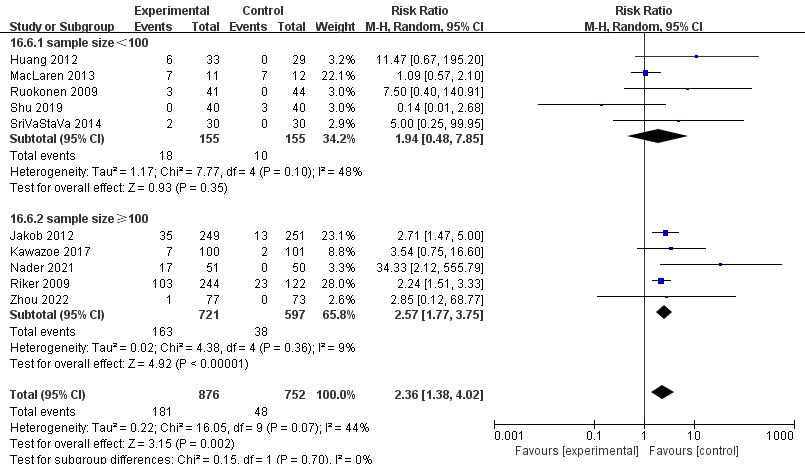


**eFigure 36.** **Hypotension Subgroup Analysis by Age of participants Forest Plot**


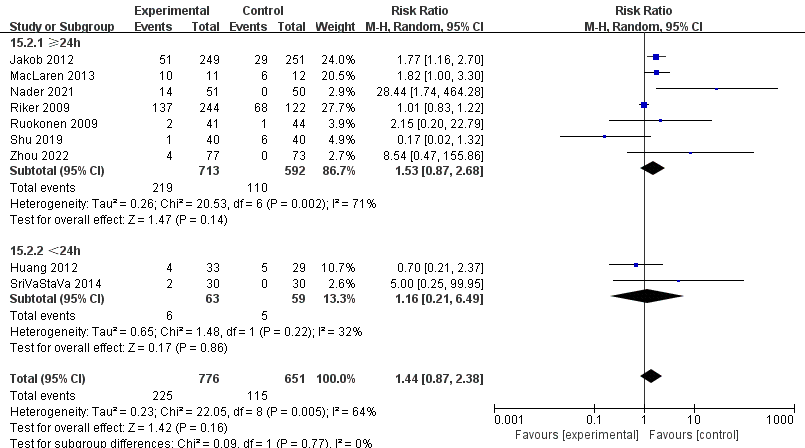


**eFigure 37. Hypotension Subgroup Analysis by Duration of sedation Forest Plot**


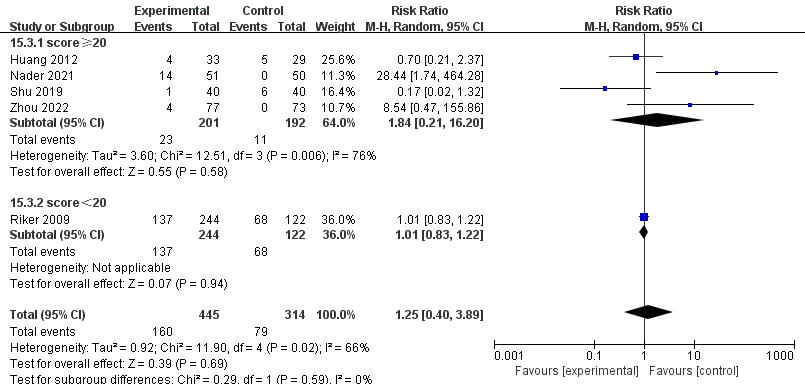


**eFigure 38.** **Hypotension a Subgroup Analysis by APACHE II score Forest Plot**


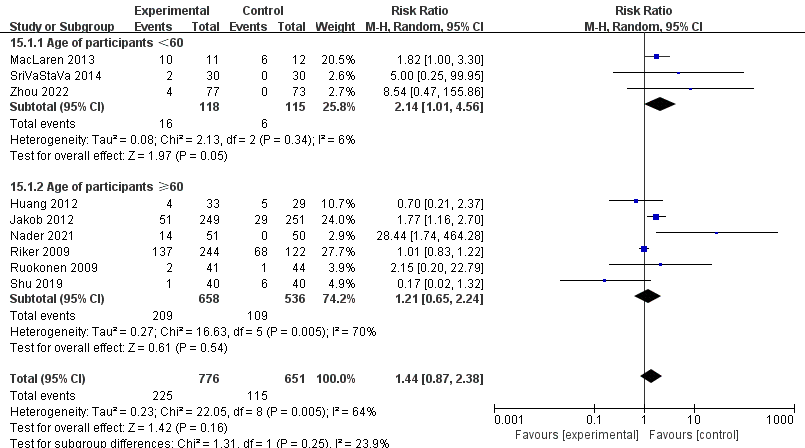


**eFigure 39. Hypotension Subgroup Analysis by region Forest Plot**


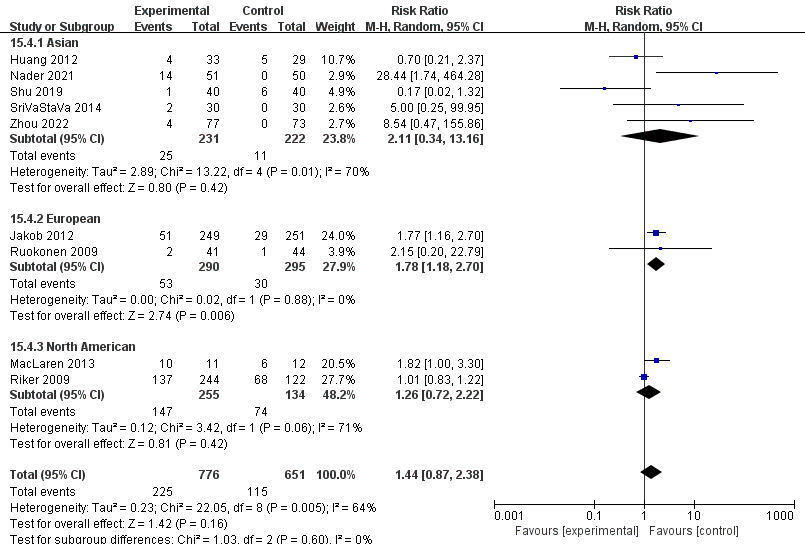


**eFigure 40. Hypotension Sensitivity Analysis by risk of bias Forest Plot**


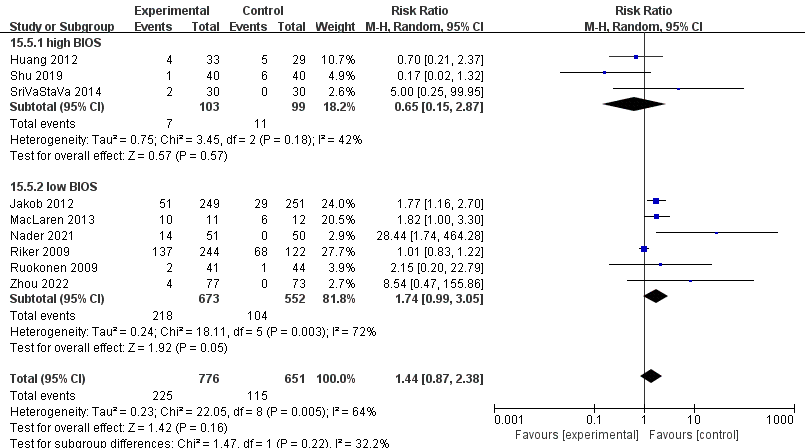


**eFigure 41. Hypotension Sensitivity Analysis by sample size Forest Plot**


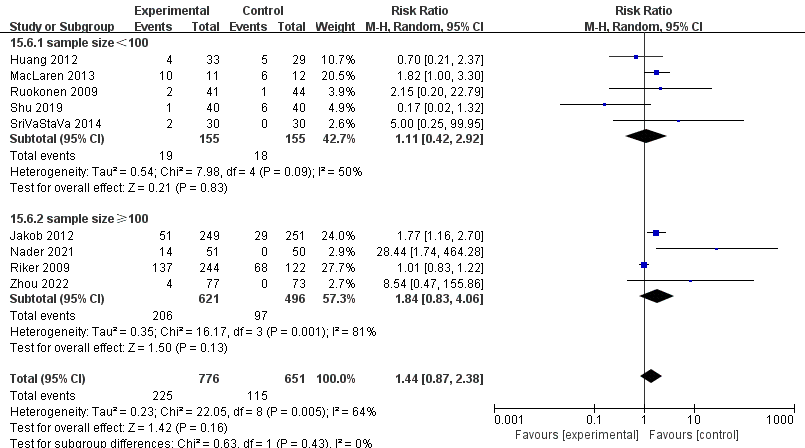


**eFigure 42. Mortality Subgroup Analysis by Age of participants Forest Plot**


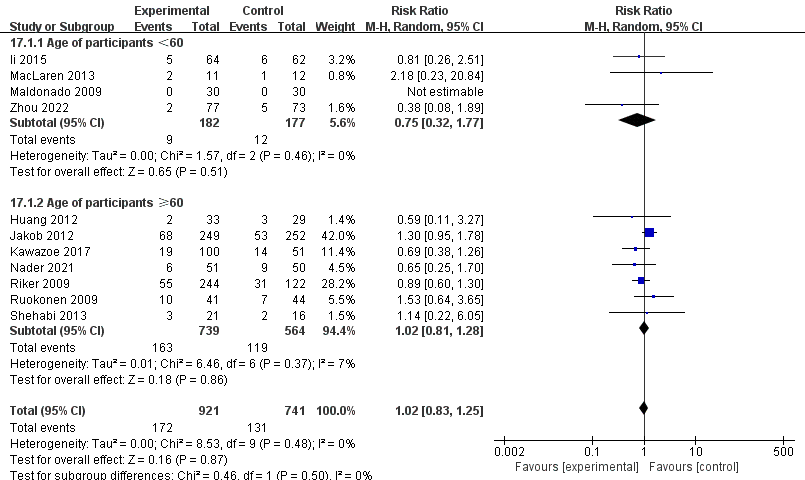


**eFigure 43. Mortality Subgroup Analysis by Duration of sedation Forest Plot**


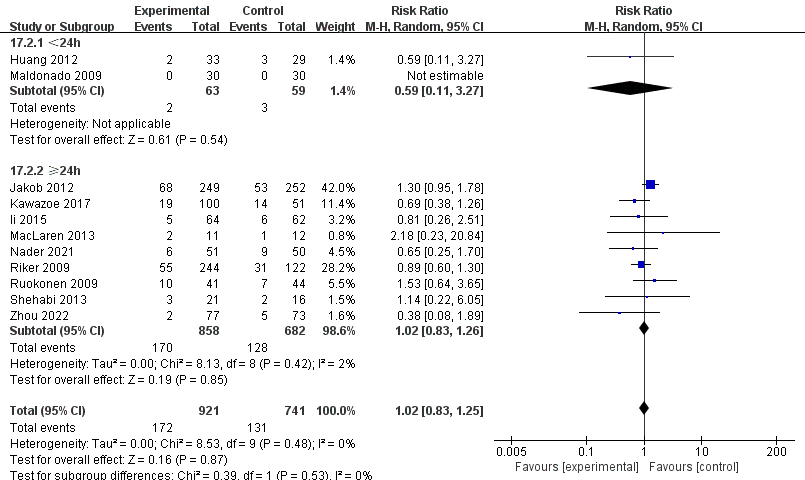


**eFigure 44.** **Mortality Subgroup Analysis by APACHE II score Forest Plot**


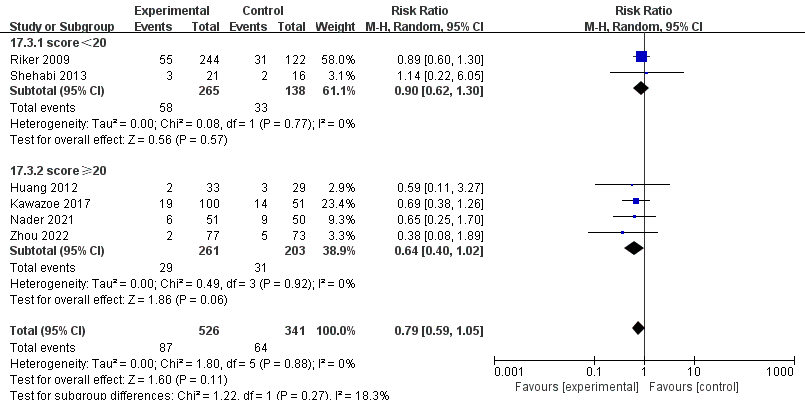


**eFigure 45. Mortality Subgroup Analysis by region Forest Plot**


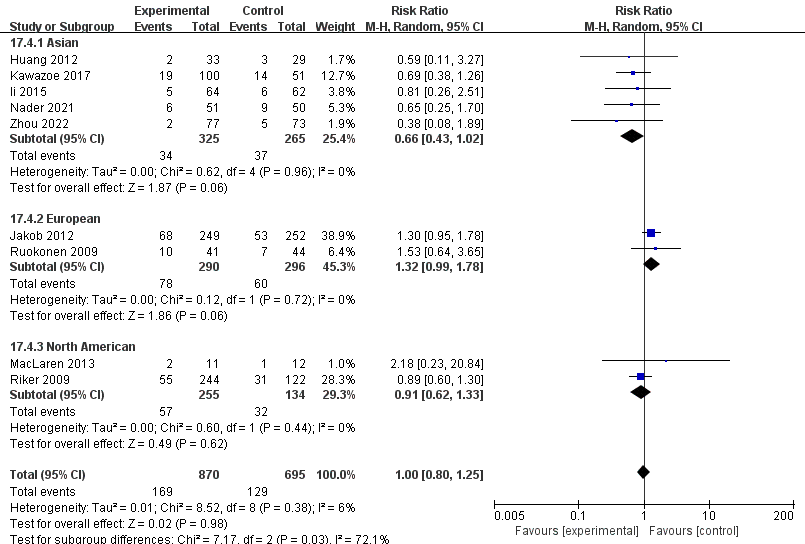


**eFigure 46. Mortality Sensitivity Analysis by risk of bias Forest Plot**


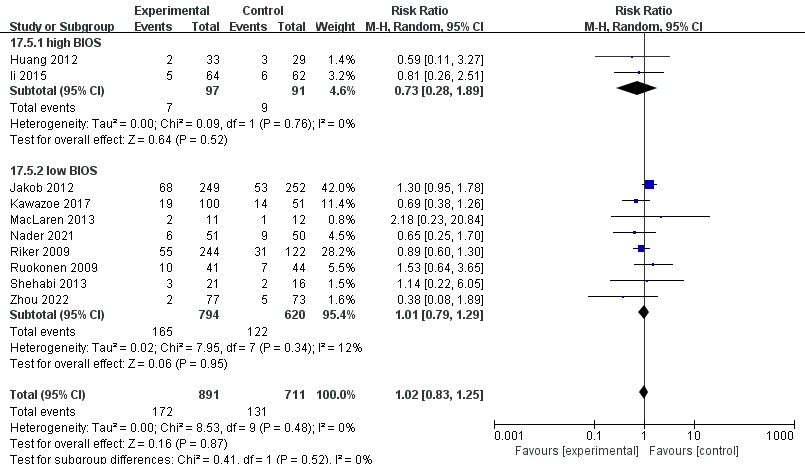


**eFigure 47. Mortality Sensitivity Analysis by sample size Forest Plot**


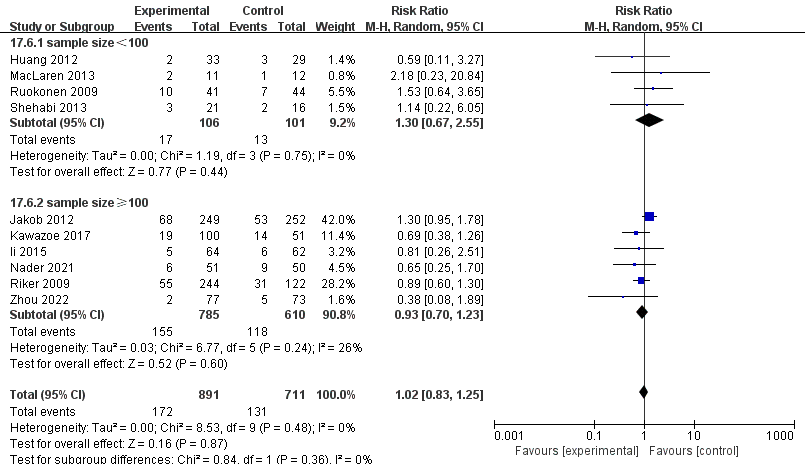

Supplement: S1 File — (DOCX) [file pone.0294292.s001.docx]
