# Supplementary material for: A comparation of dexmedetomidine and midazolam for sedation in patients with mechanical ventilation in ICU: A systematic review and meta-analysis
Source: PLoS One. 2023 Nov 14;18(11):e0294292. doi: 10.1371/journal.pone.0294292 (PMC10645332; doi:10.1371/journal.pone.0294292)
Supplement: Attachment — Submitted filename: Comment .docx [file pone.0294292.s003.docx]

***Comments to the Author:***

The authors conducted a research study titled "A Comparison of Dexmedetomidine and Midazolam for Sedation in Patients with Mechanical Ventilation in ICU: A Systematic Review and Meta-Analysis." This study addresses a critical clinical issue, and the authors have executed a well-organized and meticulously designed systematic review and meta-analysis. However, there are a few comments that need to be addressed:

Q1: The study's cutoff date was October 18th, 2022. The authors should consider performing an update of their research in accordance with PRISMA guidelines.

Q2: The exclusion criteria stating "Publications without an outcome of interest" may not be considered a valid exclusion criterion, as per the Cochrane Handbook of Intervention. The sentence should be revised to align with established criteria, such as those mentioned in the question.

Q3: In Table 1, the measurement unit for age should be included for clarity.

Q4: In Figure 1, it is advisable to provide reasons for not retrieving the two reports during the screening process. Additionally, the exclusion of 46 papers after full-text assessment raises questions. It's essential to clarify how these papers, including 22 publications that are review/meta-analyses and 15 unrelated to the research question or were animal experiments, were not excluded during title/abstract screening.

Q5: If the Forest plot is generated using RevMan, it is suggested to add labels for both groups rather than using generic "experiment/control" labels, as this can be unclear and less straightforward.

Q6: Furthermore, it is recommended to add labels for each group in the Forest plot for better clarity and understanding.
