# Supplementary material for: A comparation of dexmedetomidine and midazolam for sedation in patients with mechanical ventilation in ICU: A systematic review and meta-analysis
Source: PLoS One. 2023 Nov 14;18(11):e0294292. doi: 10.1371/journal.pone.0294292 (PMC10645332; doi:10.1371/journal.pone.0294292)
Supplement: Attachment — Submitted filename: Response to Reviewers.docx [file pone.0294292.s004.docx]

**October 17, 2023**

**Response for manuscript PONE-D-23-16182 “A comparation of dexmedetomidine and midazolam for sedation in patients with mechanical ventilation in ICU: a systematic review and meta-analysis”**

Dear Editors:

Thank you for providing us with such a great opportunity to submit a revised version of our manuscript. We appreciate your warm help with our manuscript. Meanwhile, we would like to express our sincere gratitude to all reviewers for their detailed and constructive comments on our manuscript. According to those helpful suggestions, we have revised the manuscript to make our results convincing. Revised portions are marked in red on the paper. The main corrections in the paper and the responses to the reviewers' comments are as follows.

At the same time, we ensure that the manuscript meets PLOS ONE's style requirements. The first author's ORCID ID is 0000-0002-5625-7134, and the corresponding author's ORCID ID is 0009-0009-9798-407X. we have checked all the references to ensure that they are complete and correct.

Thank you again for your time and help to our manuscript. We hope you will be satisfied with the revised version and look forward to hearing from you.

Your sincerely,

Corresponding author

Xin Liu

Encl. Responses to the comments from Reviewer 1 and 2.

**Reply to Reviewer #1**

Dear Reviewer,

Thank you very much for your time spent in reviewing our manuscript and for your encouraging comments on its merits. After careful consideration, we have further revised the article. We hope that you will be more satisfied with the revised version.

**Comments 1:**

*The study's cutoff date was October 18th, 2022. The authors should consider performing an update of their research in accordance with PRISMA guidelines.”*

**Response 1:**

Thank you very much for your affirmation of this article. Following the guidance of the PRISMA guidelines, we conducted an electronic search for recent trials to perform an update of our research.

[Relative revision can be found in the Materials and Methods part, line 73-78]

**Comments 2:**

*The exclusion criteria stating "Publications without an outcome of interest" may not be considered a valid exclusion criterion, as per the Cochrane Handbook of Intervention. The sentence should be revised to align with established criteria, such as those mentioned in the question.*

**Response 2:**

Your suggestions really mean a lot to us. we have revised our eligibility criteria under the guidance of the Cochrane Handbook of Intervention. Thank you very much.

[Relative revision can be found in the Materials and Methods part, line 84-86]

**Comments3:**

*In Table 1, the measurement unit for age should be included for clarity.*

**Response 3:**

Thanks for your valuable comments. We have added the measurement unit for age in Table 1.

[Relative revision can be found in Table 1].

**Comments 4:**

*In Figure 1, it is advisable to provide reasons for not retrieving the two reports during the screening process. Additionally, the exclusion of 46 papers after full-text assessment raises questions. It's essential to clarify how these papers, including 22 publications that are review/meta-analyses and 15 unrelated to the research question or were animal experiments, were not excluded during title/abstract screening.*

**Response 4:**

Thanks for your valuable comments. We used Endnote X9 software to remove duplicate reports. Two of the duplicate reports were not automatically deleted. When we retrieved full papers, we found these two records and removed them. After discussion, we believed that we had caused a misunderstanding, these two reports should be excluded after the records screening stage. I am ashamed that I did not catch the mistake in time. In order to improve recall ratio, we screened the references mentioned in meta-analyses and any other papers that may be relevant to our study. These articles were gotten the full text and read carefully. This led the author of the flow chart to mistakenly include these articles in the full-text assessment. According to the latest electronic search results, we have carefully revised the flow chart.

[Relative revision can be found in Results part, line 131-136 and Figure 1]

**Comments 5:**

*If the Forest plot is generated using RevMan, it is suggested to add labels for both groups rather than using generic "experiment/control" labels, as this can be unclear and less straightforward.*

**Response 5:**

Thanks for your good advice. We added the label for both groups using RevMan.

[Relative revision can be found in Results part, Figure 2]

**Comments 6:**

*Furthermore, it is recommended to add labels for each group in the Forest plot for better clarity and understanding.*

**Response 6:**

Thanks for your valuable comments. We have added labels for each group and optimized each group of figures for better clarity and understanding.

[Relative revision can be found in Results part, Figure 2-4]

Thank you very much for your affirmation and suggestions on this article. We hope the revised version will make you even more satisfied. If there is anything that needs to be modified, please do not hesitate to point it out. We are more than happy to make any further changes that improve the article.

Sincerely,

Corresponding author

Xin Liu

**Reply to Reviewer #2**

Dear Reviewer,

Thank you very much for your time spent in reviewing our manuscript and for your encouraging comments on its merits. After careful consideration, we have further revised the article. We hope that you will be more satisfied with the revised version.

**Comments:**

*Abstract:*

*length of stay in ICU should be added in the results because it is reported in the conclusions.*

**Response:**

We have added the information in the manuscript. We hope you will find this revised version more satisfactory. We are more than happy to make any further changes that will improve the article and facilitate successful publication.

[Relative revision can be found in the abstract part, line 28-34]

Sincerely,

Corresponding author

Xin Liu
